# Dual-Energy CT in Cardiothoracic Imaging: Current Developments

**DOI:** 10.3390/diagnostics13122116

**Published:** 2023-06-19

**Authors:** Leona S. Alizadeh, Thomas J. Vogl, Stephan S. Waldeck, Daniel Overhoff, Tommaso D’Angelo, Simon S. Martin, Ibrahim Yel, Leon D. Gruenewald, Vitali Koch, Florian Fulisch, Christian Booz

**Affiliations:** 1 Department of Diagnostic and Interventional Radiology, University Hospital Frankfurt, 60590 Frankfurt, Germany; 2 Division of Experimental Imaging, Department of Diagnostic and Interventional Radiology, University Hospital Frankfurt, 60590 Frankfurt, Germany; 3 Department of Diagnostic and Interventional Radiology, Bundeswehrzentralkrankenhaus Koblenz, 56072 Koblenz, Germany; 4 Department of Diagnostic and Interventional Radiology, University Hospital Mainz, 55131 Mainz, Germany; 5 Department of Diagnostic and Interventional Radiology, University Hospital Mannheim, 68167 Mannheim, Germany; 6 Diagnostic and Interventional Radiology Unit, Department of Biomedical Sciences and Morphological and Functional Imaging, “G. Martino” University Hospital Messina, 98124 Messina, Italy

**Keywords:** multidetector computed tomography, cardiovascular imaging, cardiac Imaging, dual-energy X-ray absorptiometry, dual-energy computed tomography

## Abstract

This article describes the technical principles and clinical applications of dual-energy computed tomography (DECT) in the context of cardiothoracic imaging with a focus on current developments and techniques. Since the introduction of DECT, different vendors developed distinct hard and software approaches for generating multi-energy datasets and multiple DECT applications that were developed and clinically investigated for different fields of interest. Benefits for various clinical settings, such as oncology, trauma and emergency radiology, as well as musculoskeletal and cardiovascular imaging, were recently reported in the literature. State-of-the-art applications, such as virtual monoenergetic imaging (VMI), material decomposition, perfused blood volume imaging, virtual non-contrast imaging (VNC), plaque removal, and virtual non-calcium (VNCa) imaging, can significantly improve cardiothoracic CT image workflows and have a high potential for improvement of diagnostic accuracy and patient safety.

## 1. Introduction

Since the introduction of dual-energy computed tomography (DECT), numerous technological advances led to the development of miscellaneous clinical applications based on material decomposition. In this context, calculating virtual non-contrast, non-calcium, and monoenergetic images, iodine maps for perfusion analysis using material decomposition, or the characterization of materials using labeling applications became popular. Along with tremendous efforts by scanner manufacturers to improve hardware and software, DECT regained much scientific attention and became widely available for various clinical applications encompassing all organ systems of the human body.

All of these clinical applications work on the ability of DECT to separate materials according to their atomic number. This CT technique allows material differentiation and quantification by obtaining additional attenuation measurements of the same scanned material at a second energy level. However, DECT is not only a powerful imaging modality used to obtain additional tissue information, it also better lowers beam hardening artifacts compared with conventional single-energy CT (SECT). Moreover, newer scanner generations enable rapid and essentially simultaneous acquisition of datasets at different energies resulting in substantial improvements in spatial resolution, soft-tissue contrast, and radiation dose savings.

The present review article aims to provide a comprehensive summary of hardware and software-based developments in DECT in cardiothoracic imaging and recent innovations in scanner technologies.

## 2. Basic Principles of Multi-Energy CT

Conventional computed tomography imaging is based on the principle that different types of tissues are differentiated based on their linear attenuation coefficient, which depends on the material density and composition [1]. However, as it is known that the attenuation properties calculated from a single X-ray energy spectrum are not material-specific and sometimes have similar attenuation characteristics despite different atomic structures, clear differentiation is made more difficult in certain cases. This problem can be solved using a second X-ray energy spectrum to calculate the attenuation data [2]. In contrast to conventional SECT, DECT works with two different X-ray spectra, allowing energy-dependent material decomposition by generating two datasets. The measurement of attenuation differences in images acquired at low- and high-energy spectra, with energy levels typically of 80 and 140 kVp, results in better tissue characterization. Material density and composition can easily be derived from the attenuation data of different X-ray spectra, thereby providing relevant information that cannot be obtained from single-energy CT examinations. The idea of using various energy spectra for material decomposition was initially described five decades ago by Godfrey Hounsfield, who wrote in 1973: “Two pictures are taken of the same slice, one at 100 kV and the other at 140 kV so that areas of high atomic numbers can be enhanced [1]. To date, tests showed that iodine (Z = 53) can be readily distinguished from calcium (Z = 20)” [1,3]. This essentially represents the technical basis of multi-energy CT applications (often referred to as DECT).

## 3. Scanner Technologies

The acquisition of multi-energy CT technologies is based on two different techniques: source- or detector-based (see Table 1) [2,4,5,6].

Source-based techniques include rapid spin, dual-source, rapid kilovoltage switching, and split-beam techniques [5,6,7,8,9].

Representative of detector based scanners are the dual-layer scanner and photon counting CT [10,11,12,13].

Since its early introduction in 1976, numerous technological advances allowed the development of several different CT scanners to obtain DECT datasets. In general, these DECT techniques can be categorized as a source- and detector-based, according to their primary operating principle. While dual-source, dual-spin, rapid kilovoltage switching, and split-beam technologies are source-based technologies, dual-layer and photon-counting scanners are detector-based imaging techniques [6,8,13].

### 3.1. Emission-Based Technologies

The most common is the dual-source computed tomography (DSCT) technology, which, as the name suggests, is based on two X-ray tube detector units, that are mounted on a CT gantry with an angular offset of 90° [9].

As both X-ray tubes can be controlled separately, simultaneous image acquisition with different tube voltages is possible. Dual-source DECT has a higher temporal resolution because it utilizes two X-ray sources and detectors set at different angles, allowing for simultaneous acquisition of data. This higher temporal resolution is advantageous in coronary computed tomography angiography (CTA), as it enables better capture of rapid changes in the cardiac cycle, reducing motion artifacts and providing clearer visualization of the coronary arteries during different phases of the cardiac cycle for a more accurate assessment of coronary artery disease.

One of the major setbacks of DSCT is that dual-energy information is only acquired if the dual-energy mode setting is activated by the radiographer before the acquisition. Thus, information of DECT is only acquired if actively chosen before the examination, unlike in dual-layer and photon-counting detectors, where the information is always stored and can be reconstructed if necessary after the examination, which may be helpful in accidental findings. Further, the DSCT gantry diameter is smaller due to the hardware setup of two X-ray tubes and detectors.

While the first and second generations of these systems still showed significant quality losses concerning the field of view and contrast-to-noise ratio (CNR), these problems only currently play a minor role [4,5,6].

Rapid-kilovoltage-switching scanners have only one tube detector unit, which in less than a millisecond switches between low and high tube potentials, thus enabling the recording of different energy spectra [14]. Due to the physical limitations of this technology, as well as the inherent unavoidability of a not-perfect separation of the potentials by the unavoidable transition time, this technique has physical limitations for DECT. The transition between high and low tube potentials therefore negatively affects the differentiation of energy spectra for DECT applications, as in a perfect environment we aim for perfect separation of both energy spectra. Still, an advantage is that cross-scattering or limitations in the field of view do not occur here.

Dual-spin technology requires an X-ray tube and detector layer. The scan is carried out first with a tube potential and immediately afterward with a second.

Consequently, movable anatomical structures (e.g., the heart or thoracic aorta) are unsuitable for this examination technique.

Because the two pieces of image information are acquired at different points in time, the ability to assess this can be limited because the structure to be examined is scanned in different phases of its movement [14].

The split beam or twin-beam pre-filters the X-ray beam into a low- and high-energy spectrum [8].

### 3.2. Detector-Based Scanners

Dual-layer technology uses an X-ray detector unit. Different energy spectra were differentiated at the detector level. With the help of two layers of different scintillator materials, which are sensitive to different energy spectra, real simultaneous image acquisitions of low- and high-energy data are carried out without limitations regarding field of view and cross-scattering [14]. The scan range is 4 cm on the *z*-axis, limiting the evaluation of dynamic myocardial perfusion studies. In principle, photon-counting CT requires a single X-ray tube and detector to calculate the multi-energy spectra from the attenuation caused by the examination object [10,11,15]. The SPP data record (spectral postprocessing) contains the attenuation information of each photon and thus allows the calculation of the material-typical attenuation properties. However, the NAEOTOM Alpha^®^ from Siemens Healthineers has two tube detector units.

## 4. Postprocessing

### 4.1. Material Decomposition, X-ray Absorption Spectroscopy, and Spectral Hounsfield Unit Curve

The material-specific image information is calculated based on the weighted sum of attenuation coefficients. The coefficients of a known reference material serve as the calculation differences. The ability of DECT to discriminate between different materials is derived from its dual-energy ratio (DEratio). Because DECT uses two different energies to measure attenuation, DEratio is defined as the ratio of the attenuation of a given material on a low-kV dataset to the attenuation of the same material on a high-kV dataset [9,16,17,18]. The actual characterization and differentiation between different tissue types work by correlating the attenuation of unknown tissue to known mean attenuation characteristics of commonly found materials in CT. This is possible because of the different characteristics of photon energy decrease and interaction in different tissues owing to the chemical composition. Material decomposition is achieved by utilizing the photoelectric and Compton effect. The photoelectric effect dominates at lower X-ray energies and allows for differentiating materials based on their atomic number and electron density. On the other hand, the Compton effect is more pronounced at higher X-ray energies and enables differentiation based on the effective atomic number of materials. As the energy increases, the contribution of the photoelectric effect decreases exponentially, leading to a gradual transition where the Compton effect becomes more significant for material decomposition [6,19,20]. For example, fat absorbs more energy at low energy levels, as the lipid- and water-rich composition interacts the most at low kV levels. In contrast, very dense materials, such as calcified plaques or bone material, absorb most kV levels, including higher kV. In several materials, usually used as contrast agents, a peak is visible in this curve: the so-called “K-edge,” which is specific to the atomic structure of certain elements for a certain kV level of incoming photons. The K-edge is a sudden increase in the X-ray absorption when the photon energy surpasses the binding energy of the inner-most electron shell of the atom. While the common X-ray contrast agent iodine’s K-edge is at a low energy level of ~33 kV, currently examined potential new contrast agents, such as gold (~80 kV), gadolinium (~50 kV), or bismuth (~90 kV), range in a higher kV level and might show fewer adverse effects [16,18].

Dual-energy computed tomography (DECT) utilizes various techniques to improve imaging capabilities. Rapid kVp-switching DECT, dual-source DECT, and dual-layer DECT decomposition techniques are based on two-material decomposition, while DSCT DECT adopts a different approach. Split-filter DECT enables three-material decomposition, depending on the vendor and the specific materials used for reconstruction (e.g., iodine, water, calcium, or hydroxyapatite) and application (such as fat or soft tissue) [9]. To estimate the missing information about other materials within the scanned volume, two-material and three-material decomposition algorithms utilize the acquired spectral information. Volume conservation of materials and mass conservation principles are applied. Mass conservation accuracy heavily relies on the distinct differences in the materials’ dual-energy ratios, as suggested by Liu et al. in their research [21]. In their research, they described that the accuracy of mass conservation strongly relies on the sharp difference in the dual-energy ratios of the materials. These underlying physical principles paved the way for developing several postprocessing techniques suitable for clinical use, as described below. Table 2 summarizes the benefits and potential applications of these techniques.

### 4.2. Virtual Monoenergetic Imaging (VMI/VMI+,VMC, MonoE) 

DECT allows for generating spectral Hounsfield unit curves by plotting the CT attenuation value of specific materials for different monoenergetic photon energy levels. In DSCT, this is carried out by generating monoenergetic reconstructions from two known energy levels (e.g., 80 and 140 kV), whereas in rapid kV-switching CT, this is achieved by switching the tube voltage of a single X-ray tube; for example, from 40 to 140 kV very fast. With the introduction of photon-counting detector technology, the approximation of these energy levels became obsolete, as the novel detector allows for direct differentiation of every kV level in the spectrum of the photons emitted from the X-ray tube. This means that from ~40 to 190 kV (DSCT/split filter CT), 40 to 140 (rapid kVp-switching), and 40 to 200 (dual-layer CT), we can differentiate by reconstructing the simulated attenuation levels of the materials in preset 10 kV steps [17]. While low-kV reconstructions are used for enhanced contrast, high-kV VMI/VMI+ reduces image noise and beam hardening, which is especially useful for metal artifact reduction. VMI/VMI+ cannot obliterate photon-starving artifacts from metal artifacts. VMI/VMI+ images at 40–70 keV for the evaluation of small lesions in high-attenuation low-kV reconstructions may show pseudoenhancement, which is a high artifactual attenuation observed because of a combination of beam hardening, partial volume averaging, and scatter radiation [4,6,22].

This principle can be applied to save radiation dose by using the specific attenuation coefficient of certain materials to improve their visibility. Albrecht et al. described this principle in 2019 when they investigated the specific attenuation of iodinated contrast agents in vessels for each level of monoenergetic reconstruction in DECT [23]. The result shows that low energy levels of 60 kV were optimal for visualization of the contrast agent, and further, the possibility for radiation dose reduction and reduction in contrast agent volume were discussed. This can be especially helpful in challenging clinical cases, e.g., detection of active bleeding or bad image acquisitions (missed contrast bolus) with insufficient vascular contrast in 120 kV images, because the repetition of the image acquisition may be avoided. However, as the decrease in kV leads to a significant increment of the image noise of the VMI-reconstructions, as Alvarez et al. described, this may be considered a major limiting factor to low-kV imaging for optimization of iodine contrast [24]. To avoid this issue, in DSCT imaging, there are two different data sets of two reconstructions: the virtual monoenergetic reconstruction with the lowest possible image noise and information of low-kV reconstructions with high contrast. With this, the benefit of high contrast and lower image noise can be combined, and the abovementioned issues can be partially solved [23].

### 4.3. Virtual Non-Contrast (VNC)

Especially in vascular applications, using non-contrast acquisitions is necessary to determine and differentiate between hemorrhage, contrast vessels, and other dense structures (vascular plaques, etc.). Therefore, in conventional monoenergetic CT scans, the need to acquire an additional unenhanced series often results in additional radiation doses and longer scanning times. By using post-imaging reconstructions, it is possible to calculate non-contrast series from contrasted acquisitions using DECT. These virtual non-contrast (VNC) images are virtual iodine subtraction images, which are calculated using three-material decomposition algorithms. In the literature, these artificially generated images were reported as a reliable alternative to real non-contrast acquisitions [25]. This means that even coronary artery calcium (CAC) scoring and other postprocessing and evaluations can be generated from contrast-enhanced scans. Henzler et al. reported that patients may benefit from a smaller radiation dose of up to 50% [26,27]. Particularly in emergencies, possible scan time savings may be vital. Several studies showed a good agreement between the CAC score derived from the VNC and true non-contrast CAC score scans. As a relevant limitation, incorrect subtraction of calcium content mimicking iodine contrast agents may under or overestimate calcified plaques or result in loss of information [28,29,30].

### 4.4. Iodine-/Perfusion Maps

Iodine or perfusion maps are used as a method for visualization of iodine uptake [31]. For this purpose, low-kV high-attenuation images are reconstructed and often used as an overlay “map” image overlay over an anatomical scan with high-resolution reconstruction at 120 kV. This is because iodine is particularly visible at ~40–55 kV due to the K-edge, as described above. However, in these low-kV images, the image noise is significantly high, and the images are very artifact-prone; therefore, they mainly find purpose in overlay images that are usually colorized and semitransparent with an underlying high-resolution image for orientation [32]. Furthermore, due to the iodine maps, it is possible to subtract bones and other anatomical information with high-density HU values, which may mimic iodine to the eye of the reader, thus making it easier for readers to detect bleedings and iodine uptake (e.g., tumor lesions). This can be considered a significant benefit compared to the reading of low-kV VMI reconstructions alone.

This technique is mainly used in clinical routines where ischemia is ruled out via diagnostic CT. DECT facilitates perfusion maps of the lung in the context of pulmonary artery embolism and helps to visualize perfusion deficit areas more easily. Iodine maps are particularly useful for inexperienced readers in identifying V-shaped areas of hypoperfusion in the color-coded overlay maps caused by an embolism. These V-shaped areas indicate specific regions within the parenchyma where blood flow is reduced or blocked, creating a pattern resembling the shape of a “V” [7,31,33,34,35]. Another practical application in perfusion and ischemia imaging is the evaluation of myocardial infarction during coronary CTA. This can be a beneficial tool for early ischemia detection without relying on time- and cost-intensive MRI scans for the initial first-line evaluation [29,30]. This technique has incremental clinical value and enormous potential due to optimized and more straightforward visual evaluation, which significantly increases the sensitivity for inexperienced readers, thus increasing the detection rates of pathologies associated with high mortality, such as myocardial infarction and pulmonary embolism [36,37]. 

A limitation of this technique is that it is assumed that the amount of iodine in the blood is equilibrated at the time of imaging and that the equilibrated iodine concentration remains constant throughout the scanning. In this context, iodine must be delivered through a peripheral vein at a constant rate, and the start of contrast agent injection should begin in such a way that the leading edge of equilibrated iodinated blood entirely passes through the parenchyma and back to the left heart before the spiral scan commences. This requires either a test bolus scan to determine the transit time or, with experience, one can ensure that the delay is sufficient to cover the range of individual subjects’ transit times. Kosmala et al. showed that the ideal injection protocol for the contrast medium was between 40–60 mL total with an injection rate of 5–4 mL/s, followed by a 50 mL saline chaser at an identical flow rate as the contrast agent at a concentration of 300 mg iodine/mL. Owing to improvements in detector sensitivity and the enhanced spectral separation of newer DECT scanners, it is also possible to scan without injecting a saline chaser. Injection rates of 40 mL and below with 3 mL/s were considered insufficient for good-quality iodine maps, whereas a higher amount of contrast medium was associated with a higher quality of iodine maps [38]. Bacon et al. reported a total volume of up to 100 mL (300 mg iodine/mL) [39]. In the literature, the delay varies from 7 to 12–15 s [38,40,41], and triggering thresholds are recommended to be 100–120 HU [38,39]. Considering the inter-individual differences between the patients and scanning hardware, these protocols should be an orientation for creating an ideal protocol for each site and radiographer using their experience. For the parenchymal blood volume (PBV) calculation, the first regional measurements were calibrated by dividing the regional value by the iodine signal in pure blood sampled within the pulmonary artery at the level of the pulmonary artery bifurcation. Iodine concentration highly depends on the concentration and flow of contrast media, blood pressure, and heart function. Thus, even if the protocols are standardized at a department, the concentration of iodine within the pulmonary artery will differ for each patient and will only provide a relatively quantitative result to the reader [35,37,41,42,43,44].

### 4.5. Virtual Non-Calcium

The virtual non-calcium (VNCa) reconstruction algorithm is a versatile tool with many applications for clinical use. One of the primary applications is the depiction of bone marrow pathologies such as bone marrow edema, as stated by Booz et al., Liu et al., and others [3,45,46,47,48]. By substantially increasing the image contrast of non-bone structures (bone marrow, calcified vessels, etc.), which are usually concealed in SECT by calcium mineral deposition, pathologies of these structures can be unveiled using DECT. D’Angelo et al. described many applications of VNCa in everyday life, such as traumatic injuries, inflammatory disease, infiltrative and oncological diseases, and degenerative disorders of the spine or residual skeleton in 2021. Bone marrow edema (BME) is one of the first and most important applications, as it is a biomarker of injury to the skeletal system [3,45,49].

VNCa differentiates BME from the normal bone substance by visualizing a reduction in the fat component in the trabecular bone, which is replaced by edema and hematoma. This is a significant leap for musculoskeletal CT imaging, as magnetic resonance imaging (MRI) is the only gold standard reference technique for assessing BME. Therefore, patients with pacemakers, claustrophobia, metal implants, and other contraindications to receiving an MRI examination could not receive a diagnostic image acquisition to detect BME or other pathologies not visible with conventional radiography [45].

In this setting, DECT’s ability to provide information regarding additional imaging parameters such as BME derived from VNCa images might be beneficial for a multiparametric approach to inflammatory, infiltrative, and degenerative disorders, as well as in an emergency setting for traumatized patients [3]. As CT is a prevalent diagnostic method for ruling out fractures and often a first-line imaging method for trauma patients, the application of DECT for these scans enables a one-stop-shop solution for ruling out BME, even if no fracture is found, obliterating the need for patient relocation and rescheduling. Therefore, DECT may be considered a potentially cheaper, faster, and more comprehensive imaging alternative [49]. 

In the context of oncological imaging, DECT VNCa can help differentiate hyperdense lesions from areas with high calcium concentrations in the bone, such as compact structures due to degeneration. Furthermore, DECT is applicable for collagen-based tendon and ligament imaging. This includes imaging of the cruciate ligaments, disc herniation of the spine, as published by Booz et al. in *Radiology* in 2019, and visualization of other ligaments of the body. In the context of visualizing soft tissue calcifications, direct visualization of sodium urate crystal deposition is possible using DECT. DECT is effective for monitoring gout disease activity; it was introduced as a criterion for the ACR/EULAR classification in 2015 [50].

### 4.6. Clinical Applications

#### Aortic Imaging

**Figure 1:**
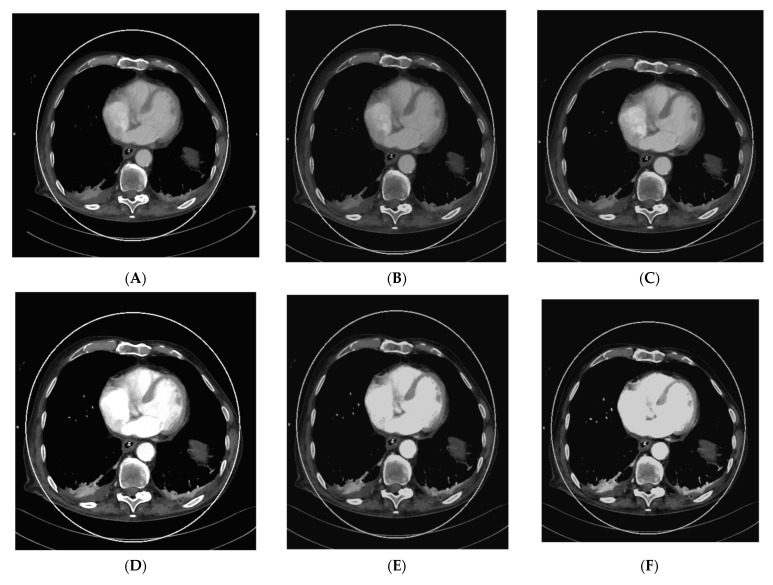

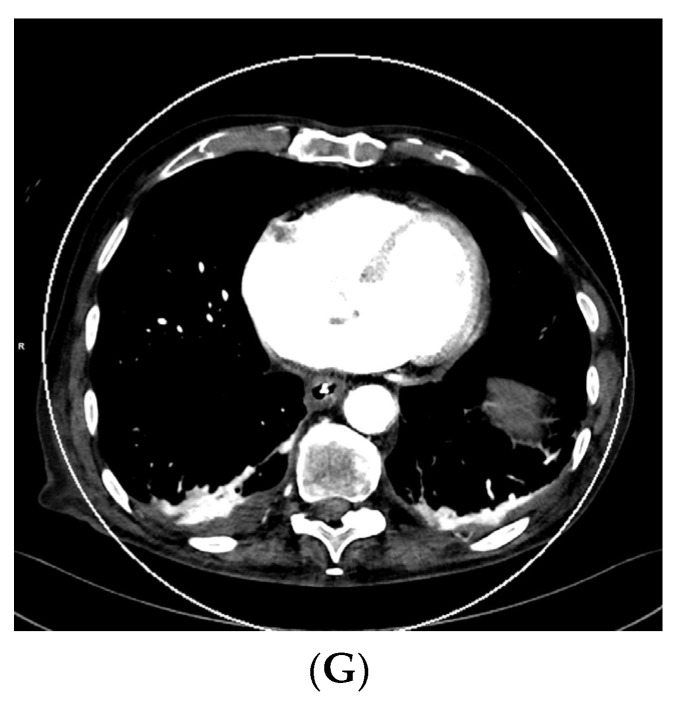
Acquisition of a DECT CTA of the thoracic aorta with a bad intravascular contrastation due to low output cardiac insufficiency in a 75-year-old male patient suffering from chest pain, tachycardia, and peripheral edema; (**A**) shows an axial view 120 kV VMI reconstruction of the descending aorta thoracalis with low intravascular contrast. Further, we see bilateral pleural effusions with adjacent partial atelectasis of the lung parenchyma. Aortic dissection could be ruled out as a cause for the clinical presentation; (**B**–**G**) show monoenergetic VMI reconstructions from 40 to 90 kV in 10 kV steps, with increasing contrast from the higher kV reconstruction of 90 kV (**B**) to the low-kV reconstructions with maximum intravasal contrast in the 40 kV reconstruction (**G**), which is closest to the K-edge of iodine, which is located at 33 kV. Accordingly, the K-edge peak is found in (**G**), as the highest intravasal attenuation is found here.

As vascular applications were one of the first fields where the benefits of DECT were demonstrated in studies and applied to routine clinical practice, aortic imaging is one of the essential fields in this context. Using DECT, it is possible to explore the effect of low-kV acquisitions using the K-edge of iodine for improved contrast in series. Martin et al. (2017), Albrecht et al. (2016), and others described that enhanced iodine contrast allows for improved detection of endoleaks; for example, in the context of thoracal or abdominal endovascular aortic repairs thoracic endovascular aortic repair (TEVAR), endovascular aneurysm repair (EVAR), fenestrated endovascular aneurysm repair (FEVAR), as well as insufficiencies and leakage of surgically implemented grafts [17,51,52,53]. Furthermore, this technique can compensate for the missed bolus and insufficient concentrations of iodine to a certain degree. The literature described that the contrast agent amount could be reduced by up to 50% in the vasculature by using 50 kV reconstructions [26]. This could be especially relevant to patients with impaired renal function, which is often associated with generalized vascular disease; however, the low-kV images also have more image noise owing to the higher rate of interaction of the photons with the atoms of the scanned body, which varies in severity depending on the DECT hardware used [6]. Figure 1 shows a case of low intravascular iodine contrast, which was caused by low-output cardiac function, as seen in the VMI reconstruction at 120 kV (Figure 1A).

Consequently, VMI reconstruction was used to optimize intravascular contrast by generating monoenergetic reconstructions from 40–90 kV in 10 kV steps (Figure 1B–G, with the maximum contrast visible in the VMI 40 kV reconstruction (Figure 1G). This is especially important in the context of the visibility of small vessels, bleeding, and low-attenuation lesions. Furthermore, iodine mapping can be used to prove the accumulation of extravasal iodine in hematomas and bleeding. Using mixed images, for example, 120 kV, which is calculated from the high- and low-kV images, can improve the image noise. Furthermore, it is possible to create virtual unenhanced images or use other material-specific applications that are derived from (virtual) monoenergetic images. These monoenergetic images can be reconstructed at 40–190 kV for DSCT DECT. In this context, calcium subtraction from calcified plaques is important [17,20].

As the images are all reconstructed from the same raw dataset, it is also possible to use subtraction and fusion techniques. A 2:1 ratio of high-kV images to low-kV images was used for composite images, as the noise in the low-kV images significantly influenced the overall image noise [6].

The most common combination of tube voltages is 140 and 80 kV, or 150 and 80 kV for dual-source DECT systems and 135 and 80 kV for single-source fast-kV switching CT. If more low-kV data are used, the problem of image noise can be reduced by modern reconstruction techniques, such as nonlinear sigmoidal blending of low- and high-peak kilovoltage data and deep learning-based processing of the raw data. The optimum voltage for visibility of the iodine contrast agent in fast-kV switching is approximately 55–60 kV [6].

The high kV range is optimal for metal artifact reduction and the visualization or subtraction of calcified vascular plaques [54].

In this context, one of the most significant benefits of DECT in vascular imaging is the reconstruction of virtual non-contrast (VNC) images, as it allows for better visualization of intramural hematoma, thrombosis, and dissection diagnosis. As a normal unenhanced CT scan is usually a standard acquisition for aortic imaging protocols, this technique can substantially reduce the radiation dose, particularly in patients undergoing regular follow-up examinations. However, the image noise of VNC images is higher than that of real unenhanced images but is of diagnostic quality in >95% of cases, owing to recent software and hardware developments [25]. VNC images, which can be retrospectively reconstructed, are a great asset in cases of unexpected findings (such as bleeding, tumor mass, and inflammation). The limitation of this technique is that there might be an overcorrection of iodine uptake subtraction of opaque structures close to iodine-rich structures in acquisitions, such as the underestimation of small calcified plaques [54,55]. 

This is especially relevant as complex protocols are required for specific examinations and clinical indications (such as endovascular aortic repair EVAR, TEVAR, and FEVAR); in literature, many different approaches are described, from multiphase CT with arterial venous and unenhanced images to dynamic CT acquisitions different protocols were discussed. When multiphase or even dynamic acquisitions are performed, the radiation exposure can be very significant. Thus, this field is an ideal application for VNC reconstructions in the context of endoleak detection. In the context of aortic imaging and visualization of pathologies, VRT 3D reconstructions with automatic parenchyma and bone subtraction are frequently reconstructed and well-accepted examples for the applications of DECT [54]. In these 3D models, all image data are reconstructed, but the information about the vessels is used to create a 3D model of the vessels and branches and plaques, prosthetics, and stents that might be present. Clinicians often prefer viewing 3D reconstructions over axial images because they resemble the situs during surgery. Bone subtraction in single-energy images is usually performed by thresholding or seed point-growing techniques, or as of lately, AI learning algorithms, which are still very difficult and time-consuming to achieve good results, as small structures such as ribs are difficult for the software to detect. Therefore, in conventional 3D subtraction techniques, there is often a loss of information, particularly in the visualization of small vessels and side branches. It is possible to achieve faster and better 3D reconstruction results using DECT, particularly in the context of vascular diagnostics. In this context, creating contrast-colored overlay maps can be helpful in highlighting iodine uptake or endoleaks.

### 4.7. Pulmonary Embolism

In the context of pulmonary embolism, DECT imaging has many benefits for clinical use. A good acquisition of a CT scan to rule out pulmonary embolism can sometimes be challenging due to the timing of the bolus and the acquisition of an optimum contrast in the pulmonary arteries. Pulmonary artery contrast can also be negatively affected by patients contracting abdominal muscles while holding their breath for the examination, thus performing a vasovagal maneuver leading to the contrast agent being pressed retrogradely from the right heart into the superior vena cava. Kosmala et al. described insufficient contrast of the pulmonary arteries in some patients even though the bolus timing was estimated correctly [38]. Furthermore, detecting smaller segmental areas of hypoperfusion and embolisms can sometimes be difficult, especially for residents and trainees [4,22,43]. Zhang et al. and other research groups described that DECT offers many possibilities to compensate for non-optimal image acquisitions or support the visibility of pathologies by its ability to create virtual monoenergetic images that enhance the contrast of iodine (one of the most used is the 55 kV reconstruction) to compensate for low contrast in the pulmonary arteries [38,44]. 

**Figure 2:**
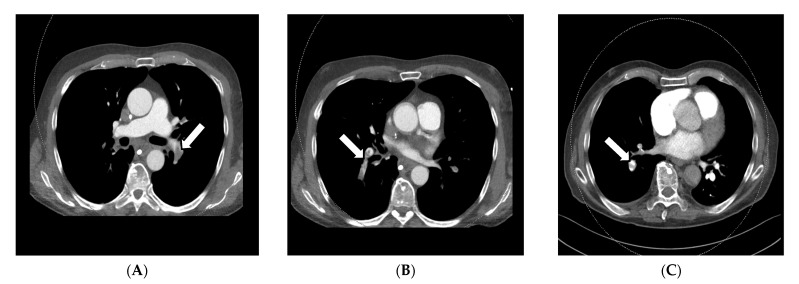

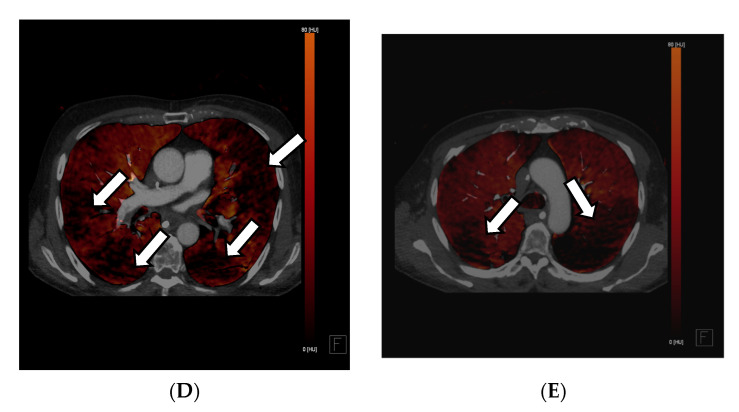
Application of iodine mappings and perfusion visualization facilitating DECT in a DSCT scan of a 23-year-old woman suffering from chest pain, tachycardia, and dyspnea. The 50 kV axial soft tissue kernel reconstruction CT angiography of the pulmonary arteries showed central thrombosis of both main pulmonary arteries on the right side (**A**,**B**) and the left side (**C**) with partial occlusion of the subsequent vessels. V-shaped perfusion deficits were reported in the perfusion maps (**D**,**E**), which can be correlated to segments 9/10. In correspondence to the missing contrast agent in the responding segmental arteries of these segments, a total occlusion was suspected. The patient was accordingly treated with systemic anticoagulation.

For diagnosing pulmonary embolism, iodine mapping, and perfusion analysis are advantageous features for the visualization of hypoperfused areas, usually V-shaped areas in the perfusion maps, which can be used as a colored overlay map on anatomic CT reconstructions according to Thieme et al., 2008 and many other publications. This semi-quantitative technique allows for relative measurements of iodine uptake in the lung parenchyma (See Figure 2A–D) [31,33,34,37,38,41]. 

A potential limitation of angiographic imaging in the thorax is that, with first-generation DSCT, the 26 cm FOV may limit bone subtraction outside the central FOV. These limitations are significantly reduced by the 33 cm FOV of second-generation DSCT and, theoretically, are not an issue with single-source acquisition techniques [6].

### 4.8. Cardiac Imaging

One of the most critical applications of DECT is cardiovascular imaging in coronary artery angiography (CCTA) and DECTs for detecting parenchymal pathologies, as stated by De Santis et al., 2018 [29].

To date, CCTA is a non-invasive method for diagnosing coronary atherosclerosis and plaque burden, which was proven to be as sensitive and specific as invasive conventional coronary angiography. It is a feasible means for primary diagnostics and follow-up. Usually, a non-contrast scan is used to evaluate calcified atherosclerotic plaque burden according to the Agatson score to evaluate the severity of coronary disease [28,56].

Due to DECT techniques such as VNC, it is possible to calculate reliable non-contrast images, which have a similar diagnostic value compared to real/true non-contrast (TNC) images. This significantly reduces the radiation dose by saving one additional acquisition and optimizing the scan times, as described by Holtz et al. in 2020 and Van Assen et al. in 2021 [25,55]. With virtual non-calcium (VNCa) applications, it is possible to remove calcified plaques from images, thus optimizing the evaluation of the residual lumen in CCTA [25].

**Figure 3:**
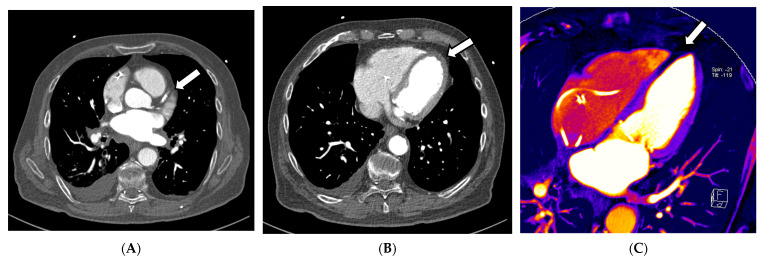

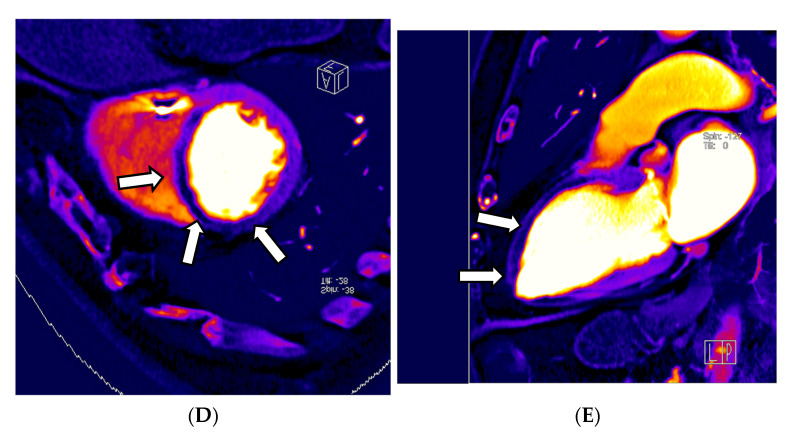
Exemplary cardiac application of DECT in a patient admitted to the emergency chest pain unit suffering from acute angina pectoris symptoms with troponin and CK-MB elevation. The acquisition of a 120/80 kV third-generation dual-source DECT showed occlusion of the right interventricular artery (RIVA) in the CCTA (**A**). In the axial soft tissue kernel reconstruction, the interventricular septum and the myocardium of the left ventricle showed mild hypodensity at a closer look (**B**). Additionally, iodine perfusion maps were calculated from the data, revealing a myocardial perfusion deficit of the anterolateral wall and parts of the anterior septum (**C**–**E**). These findings correlate to signs of an anterolateral myocardial infarction, also reported in the ECG in the emergency room.

In this context, in addition to common CCTA, additional information on myocardial perfusion imaging (MPI), and thus, the functionality and prognosis of myocardial infarctions, can be derived according to Jin et al. (2016) [36]. This information can be derived from iodine images or maps, which are surrogate parameters of organ perfusion, by visualizing the amount of iodine from the contrast agent present. Kwan et al. (2021) describe that if a regional lower iodine attenuation is visible in the myocardium, it indicates myocardial infarction [36,57]. This is consistent with the method of creating iodine maps to indicate arterial lung embolism, in which the quality of DECT is essential, as the accuracy of iodine maps is directly related and proportional to the DE iodine, which is calculated by comparing the attenuation values of iodine at the two different energies. This ratio provides information about the iodine concentration within the scanned region. This means that the better the spectral separation of the high- and low-kV spectra, the less noise occurs, while the image quality of iodine maps improves, and likewise, the better the VNC images are that are created from the data. These two techniques, CCTA and myocardial perfusion, play a significant role in cardiovascular diagnostic imaging; some approaches combine both in one examination (see Figure 3A–D). Despite very promising results regarding specificity and sensitivity, false-positive CCTA MPIs can occur, which might be explained by possible artifacts from beam hardening or high iodine quantities in the heart, causing inaccurate perfusion maps [58]. As discussed above, the quality of the DECT examination is responsible for achieving a good DE iodine ratio. If the scan protocol, hardware, or patient positioning in the central FOV is insufficient, this can severely impact the image quality, thus affecting the reliability of the perfusion images, which limits the impact of MPI for clinical usage. According to Albrecht et al. (2018), the findings should be cautiously assessed and related to each patient’s clinical symptoms, similar to hybrid positron emission tomography and SPECT/CCTA studies. Still, it was proven that coronary artery CT of the heart using the latest generation MDCT has a diagnostic accuracy similar to that of invasive coronary angiography [58]. For the detection of chronic and acute abnormalities of the coronary arteries, an ECG-triggered CT of the heart can be highly accurate; however, such as in any CTA, the contrast can be insufficient due to several issues. One reason may be the insufficient concentration of iodine contrast in the vessel at the acquisition time. It can be an additional problem in systems with small detectors, which must acquire 2–3 different CT volumes in the diastole of heart action. The acquisition of a combined CT scan can take up to three heartbeats; thus, a short bolus can lead to missing the maximum contrast concentration in all or several volumes, leading to a decreased diagnostic validity of the study. In the context of coronary atherosclerosis, even small plaques can cause a difference in the perfusion of the myocardium. In conventional cardiac CT, blooming artifacts can lead to over- or after-subtraction techniques, even to underestimation of the obstruction of the lumen [57].

In the CADRADS 2 criteria for diagnosing coronary artery disease, four vulnerability criteria were described (low attenuation, positive remodeling, spotty calcification, and the napkin ring sign) [59]. These signs, directly correlated with a potential risk of myocardial ischemia, lead to an upgrade in the classification. Thus, it is clinically relevant to have a cardiac CT with high sensitivity and specificity [28,30]. 

In examinations where image quality is crucial for accurate diagnoses, such as CTA of the coronary arteries, movement artifacts are one of the biggest challenges. As the heart’s movement only pauses for a short time during diastole, which is when the acquisition of the CT scan takes place, a shorter diastole implies a shorter acquisition time. Therefore, the longer the pauses between the heartbeats, the better the CT scan’s image quality. As a result, tachycardia and arrhythmia are among the most common errors leading to insufficient and sub-diagnostic image quality, as described by Albrecht et al. (2018) [58]. 

Patients may receive oral or i.v.-injected beta-blocker medication before image acquisition to slow down the heart rate, aiming to reach a frequency of <65 bpm. Of course, this is not possible for patients with contraindications and arrhythmia and is often unnecessary in patients with normal frequency and sinus rhythm. Further, Schmidt et al. (2020) described recent advances in CT hardware with dual-source DECT to achieve shorter acquisition times and gantry rotation times of up to 0.25 s at 0.6 mm slice thickness in 90° angulated DSCT, and a pitch of 3.2 (twice as high as single-source CT), which have a temporal resolution of ¼ of the rotation time [6]. This is possible because coronary artery CTA (CCTA) reconstructions of 180° scan segments plus the fan angle of the detector can be used (50–60°, depending on the vendor) to create a full scan field of view images ~50 cm in diameter. This leads to a very high temporal resolution without using segmental reconstruction techniques and sharp images, even with heart rates above 65 bpm. Particularly in patients with atrial fibrillation or arrhythmia, DECT CCTA was found to be more precise and has a high diagnostic value. In contrast to dual-DSCT DECT, single-source systems require a larger detector to cover the whole heart volume; thus, the pitch is limited to <1.5, as otherwise, sampling gaps occur, and thus, loss of information and artifacts [6]. 

Schmidt et al. described that a problem with DSCT, which occurs during DECT acquisitions, is that owing to the small gantry, the organ of interest has to be in the central FOV of both detectors, which vary in size (e.g., 26 cm vs. 50 cm FOV). This is due to the hardware design; therefore, data outside the FOV of the smaller detector must be extrapolated from the reconstruction, as otherwise, they may lead to data truncation artifacts [6]. In the context of DSCT for DECT imaging, the aim is to obtain the energy spectra of both tubes as well separated as possible to avoid overlaps in energy levels that lead to optimized dose efficiency and fewer beam hardening artifacts. Parakh et al. reported that to achieve the best possible filtration of the non-usable energy spectra, tin and copper filtration is used [22]. Another benefit of DECT in vascular imaging is that atherosclerosis and stent imaging can be vastly improved by optimizing imaging artifacts. Common artifacts include blooming artifacts, which lead to overestimation of calcified plaque size due to reconstruction techniques that try to smooth the images; by doing so, regions where very high-density objects (calcified plaques) and low-density objects (blood in the vessel lumen) are mixed by binning techniques that aim to reduce image noise. In this context, the use of rapid kV-switching DECT showed benefits in some studies, which evaluated the diagnostic accuracy of in-stent stenosis, while some authors reported stronger artifacts using kV energies of approximately 80 kV. This may be avoided by sharper filter kernels or wider window and iterative reconstruction techniques, as well as higher spatial resolution scanners (e.g., photon-counting CT) and DECT techniques [57]. DECT facilitates tissue decomposition using spectral imaging information to differentiate between calcified and atheromatous plaques and physiological lumen. The result is a sharper identification of calcified plaques and reduced overestimation of atherosclerotic plaques. In particular, high-kV monoenergetic reconstructions with sharp filter kernels provide a better differentiation between plaque and lumen and reduce the overestimation of plaques or metal artifacts [28]. In 2021, Chen et al. published a paper on DECT reconstruction techniques that cover limited angular ranges (LAR) using a directional total variation algorithm; thus, images can be reconstructed with scan angles of 14–180° with reduced artifacts and better image quality [60]. 

### 4.9. Spine Imaging

Diagnosing cervical degeneration of the spine, osteoporosis via bone densitometry, and imaging of traumatic injuries is very important in clinical practice. DECT facilitates several applications to improve the diagnostic value of CT scans for these indications [3,61]. In this context, Booz et al. described the significant benefits of DECT for the evaluation of vertebral degeneration, especially in the cervical spine, which often leads to severe symptoms due to spinal or neuroforaminal stenosis, often caused by a combination of osseous and discoligamental pathologies. Therefore, the quality requirements for spine imaging are very high, which is often a cause of MRI acquisition. However, with the implementation of DECT, it is possible to use the high spatial resolution of CT and provide good visualization of vertebral discs and ligaments [3,46]. Booz et al. reported excellent sensitivity and specificity compared to MRI as the gold standard for disc herniation in the cervical spine and 95% confidence intervals of 91–98% for unenhanced DECT [48,62], which was confirmed by Shim et al. in 2022 with sensitivity levels of 94% and specificity of 90% [63]. In 2022 Koch et al. also described excellent sensitivity of 94% and a specificity of 95% compared to MRI in portal venous phase DECT in thoracic disc herniation [64]. 

**Figure 4:**
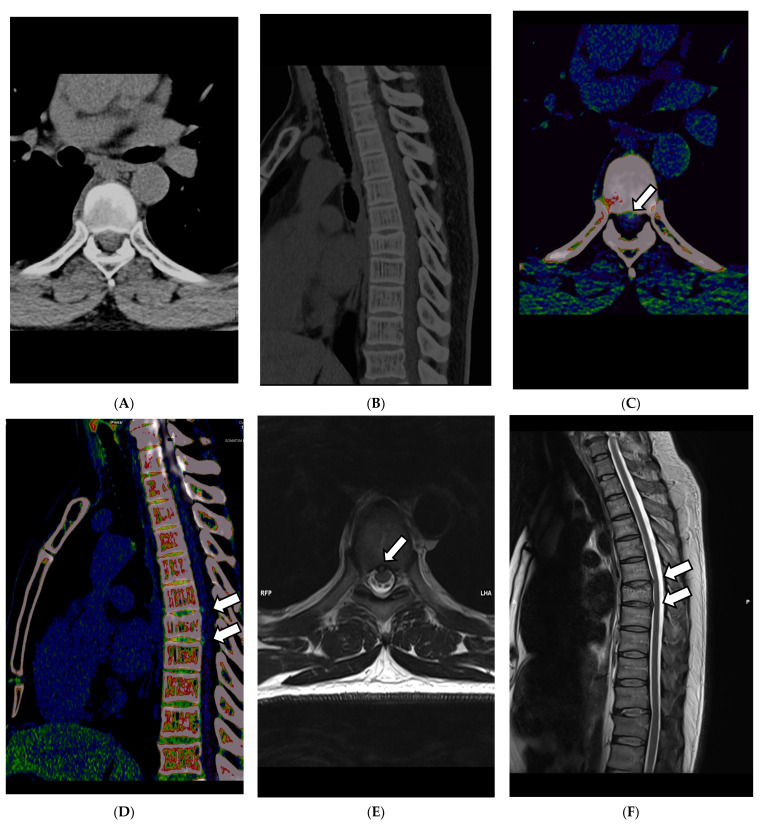
DECT scan of the thoracal spine in a 61-year-old female patient that presented herself with focal pain at the upper back in the clinical examination after slipping and falling on her back. The clinicians suspected a possible vertebral fracture. Thus, a CT scan without a contrast agent was scheduled. During the reading of the images, fractures were ruled out quickly. In the VMI 120 kV soft tissue kernel CT reconstruction, as shown in (**A**) axial and (**B**) in sagittal orientation, disk herniation was hardly visible. Application of color-coded VNCa DECT reconstructions finally revealed dorsal median disc herniations (indicated by the white arrows in (**D**,**F**)) in segments T 6/7 and T 7/8, as seen in (**C**) in axial orientation and (**D**) in sagittal orientation. The finding was later verified in a 1.5 Tesla MRI examination, where the disc herniation could be correlated to the CT findings, as seen in the axial T2-WI sequence (**E**) and the sagittal T2-WI sequence (**F**).

Applications such as color-coded VNCa enable improved visualization of disc herniation (see Figure 4A–D). In 2021, Koch et al. evaluated the diagnostic sensitivity, specificity, and accuracy of color-coded VNCa for the detection of thoracic disc herniation. They found that DECT was significantly better in the tested categories than conventional CT, with a sensitivity of 95% in DECT vs. 73% in SECT and a specificity of 96% in DECT vs. 82% in SECT [64].

Another helpful feature of VNCa is the possibility of detecting bone marrow alterations in trauma CT scans of the spine, as described by Booz et al. in *Radiology* in 2019 and as described by Fevres et al. in 2022, which are usually not readily visible in conventional CT. After severe trauma to the spine, it is common to use CT acquisition to rule out fractures of the cervical spine, which are barely visible in conventional radiography [45,65,66,67]. By subtraction of calcium from the bone, differences in the bone marrow are depictable, such as hematoma of the vertebral body, in a color-coded overlay, which will be added as an overlay map over the 120 kV anatomical reconstruction of the CT images. This leads to significantly improved detectability of BME with a sensitivity reported by Cavallaro et al. of 89% and a specificity of 98% [45,49,66]. In 2022, Gruenewald et al. reported a very high potential for VNCa as a predictive mean for the volumetric evaluation of bone mineral density (BMD) for the 2-year occurrence risk of osteoporosis-associated fractures, with 85.45% sensitivity and 89.19% specificity [61]. All the recent developments in this field are highly promising for trauma imaging and workflow improvement by enabling a sensitive method for detecting pathologies and gaining information usually only accessible for patients undergoing MRI acquisitions, which are often not accessible in an acute clinical setting. Consequently, DECT facilitates an excellent potential for time and cost savings in departments with limited access and promotes responsible use of diagnostic means and resources.

## 5. Limitations

DECT emerged as a promising imaging modality in cardiothoracic applications, providing enhanced tissue characterization and improved diagnostic accuracy. However, there are limitations to this technique that need to be addressed. Similar to common SECT, beam hardening artifacts represent one of the primary limitations of DECT in cardiothoracic imaging. These artifacts occur due to tissues’ differential energy absorption properties, resulting in image distortions that can compromise diagnostic accuracy, e.g., the presence of metal artifacts, particularly in patients with metallic implants or devices. Metallic objects can cause streaking, blooming, and dark band artifacts, degrading image quality and obscuring critical anatomical structures (e.g., patients with cardiac pacemakers). The metal artifacts around the device can hinder the visualization of adjacent cardiac structures, limiting the accurate evaluation of potential cardiac abnormalities. For example, beam hardening artifacts may obscure the visualization of calcified plaques in the evaluation of coronary artery stenosis, leading to potential underestimation of the disease severity. While higher kV reconstructions can reduce these artifacts in this context, low-kV reconstructions may even aggravate the artifacts. Thus, the choice of the right monoenergetic reconstruction level is crucial.

Different hardware approaches employed by different vendors in DECT systems can impact the temporal resolution, which is particularly relevant in the context of coronary CTA. Insufficient temporal resolution can lead to motion artifacts and reduced image quality, affecting the accurate assessment of coronary artery disease. Clinicians and researchers must be aware of these hardware-related limitations and consider them when selecting and interpreting DECT images for coronary CTA.

Another limitation is that DECT utilizes two different energy spectra, which requires acquiring two image sets. Thus, radiation dose must be considered, and protocol adaptation has to be performed not to exceed SECT dose limits. A combination of hardware and software optimization strategies can be implemented. Hardware optimization includes tube current modulation techniques, advanced detectors, and iterative reconstruction algorithms. Software optimization involves selecting appropriate tube voltage, utilizing dose reduction techniques, and optimizing DECT protocols. These measures collectively minimize radiation exposure while maintaining the image quality and diagnostic accuracy of low-dose DECT.

## 6. Discussion and Conclusions

Since its introduction more than 50 years ago, computed tomography (CT) became an indispensable diagnostic tool in clinical practice. However, magnetic resonance imaging (MRI) is preferred for many clinical issues due to its better discrimination of soft tissues, among many other advantages.

Technological improvements in recent decades resulted in faster scan speeds, greater scan volume coverage, and higher temporal resolution. One of the most recent challenges is the increasing use of CT in routine clinical practice while maintaining an appropriate radiation dose for patients. In addition, functional and spectral CT with dual- and multi-energy applications and perfusion imaging is increasingly used in routine clinical practice. Technically difficult images such as those of the heart and aorta benefit significantly from these innovations, allowing even less experienced radiologists to achieve a high level of diagnostic confidence for clinically relevant issues, such as lung embolism, myocardial infarction, and aortic dissection. This massively increased the diagnostic quality of cardiothoracic CT and even extended it to use cases that were previously the exclusive domain of MRI examinations, such as bone marrow edema after trauma. As novel photon-counting detectors are being installed in increasing numbers worldwide, the possibility of high spatial resolution and spectral imaging is opened for every examination and patient, regardless of the CT protocol. 

## 7. Future Directions

Modern computed tomography is experiencing a renaissance thanks to parametric imaging techniques such as dual-energy CT, and it is regaining its importance by enabling fast, pragmatic, individualized, pluripotent, and precise diagnostic imaging pathways for cardiothoracic imaging. Novel postprocessing algorithms and artificial intelligence-based data analysis are progressively increasing the diagnostic value for each CT scan in most cases without additional radiation exposure to patients. 

We are witnessing the beginning of a new era of cardiothoracic imaging using spectral dual-energy CT and novel photon-counting CT, which is becoming increasingly important and used in routine clinical practice. Both patients and clinicians are already and will incrementally benefit from these developments in the near future.

## Figures and Tables

**Table 1:** Table of relevant dual-energy computed tomography (DECT) hardware, comparing hardware and postprocessing related system information between vendors and scanner types. Field-of-view (FOV), virtual mono-energetic imaging (VMI). Z-cover represents the longitudinal coverage of the CT detector in the *z*-axis of the patient.

Manufacturer	Philips	GE	Canon	Siemens	Siemens	Siemens	Siemens
Commercial Name	IQon Spectral CT	Revolution	Aquillion ONE Prism Edition	SOMATOM Definition Force	SOMATOM Definition Flash	SOMATOM Definition Edge	Somatom Drive
DECT principle	Multi-layer energy sensitive “sandwich detector” Detector based	Rapid kV-switching	Rapid kV-switching	Dual-source Dual-energy	Dual-source Dual-energy	Twin-beam Dual-energy	Dual-source Dual-energy
FOV DECT/low voltage X-ray tube [cm]	50	50	50	35,6	33	26	48
Radiation filtration in DECT	Not available	Not available	Not available	Sn filter	Sn filter	Sn filter	Sn filter
Z-cover (mm)	40	160	160	115.2	64	160	131
VMI reconstruction range	40–200	80/140 kV, 40–140 kV	80/135 kV, 35–200 (applikation)	40–190	40–190	40–190	40–190
Detector rows	2 × 64	250	320	2 × 192	2 × 128	2 × 64	2 × 128
Measured slice thickness (mm)	0.6	0.6	0.5	0.6	0.6	0.6	0.6

**Table 2:** Table containing clinically relevant dual-energy computed tomography (DECT) postprocessing techniques and clinical applications based on the material decomposition techniques and spectral information acquired by DECT scanners. Lung–artery embolism (LAE); coronary computed-tomography angiography (CCTA).

Technique	Clinical Applications	Advantages
Virtual Monoenergetic Imaging (VMI/VMI+) High-kV	Visualization of metal implants, beam hardening artifact reduction.	Beam hardening artifact reduction, in stent visibility, and reduced image noise.
Virtual Monoenergetic Imaging (VMI/VMI+) Low-kV	Vascular: missed contrast bolus or poor intravasal contrast (rule out of LAE during CCTA).	Improved contrast and visualization of iodine, bleeding detection.
Virtual non-Contrast (VNC)	Cardiac CT, vascular and multiphase CT-protocols.	Radiation dose reduction, reduced scan time, and improved workflow.
Iodine mapping	Myocardial perfusion, pulmonary embolism perfusion maps.	Improves sensitivity and detectability for iodine and bleeding, and quantifies iodine uptake and perfusion information.
Virtual non-Calcium (VNCa)	Trauma imaging, bone marrow edema, bone mineral density (BMD) quantification, and disc herniation.	Additional information on bone marrow edema and occult fractures without MRI, improved workflow and sensitivity.

## Data Availability

Not applicable.

## Abbreviations

CAC	Coronary artery calcium
CADRADS	Criteria for diagnosing coronary artery disease
CCTA	Coronary computed tomography angiography
CT	Computed tomography
CTA	Computed tomography angiography
DECT	Dual-energy computed tomography
DEratio	Dual-energy ratio
DSCT	Dual-source computed tomography
LAR	Limited angular ranges
MDCT	Multidetector computed tomography
MPI	Myocardial perfusion image
NPV	Negative predictive value
PCCT	Photon-counting computed tomography
PPV	Positive predictive value
SECT	Single-energy computed tomography
SPP	Spectral postprocessing
TNC	True non-contrast
VMI/VMI+	Virtual monoenergetic imaging
VNC	Virtual non-contrast
VNCa	Virtual non-calcium
